# Comforting strategies and perceived barriers to pediatric pain management during IV line insertion procedure in Uganda’s national referral hospital: A descriptive study

**DOI:** 10.1186/s12887-015-0438-0

**Published:** 2015-09-16

**Authors:** Godfrey Katende, Benedicto Mugabi

**Affiliations:** Sultan Qaboos University, College of Nursing, 123 Muscat, Oman; Department of Nursing, Makerere University,College of Health Sciences, 256 Kampala, Uganda

**Keywords:** Comforting strategies, Health care provider, Pain management, Pediatric

## Abstract

**Background:**

Venipuncture and intravenous (IV) cannula insertions are the two common sources of pain in hospitalized children and health care today. The WHO asserts that, pain relief is a basic fundamental right and requires a multidisciplinary approach. Nonpharmacological comforting strategies when implemented are important to relive pain related distress in children during peripheral IV line insertion. However, evidence to date that suggests implementation of such strategies and their barriers in Uganda remains very limited. This study aimed at establishing the current practices in regard to the use of comforting strategies and the perceived barriers faced by health care providers to implement pediatric pain management during IV line insertion procedure in Uganda’s national referral hospital, Mulago.

**Method:**

A cross sectional and descriptive study was conducted between December 1, 2012 and February 28, 2013 involving doctors, nurses and interns in six pediatric wards of Mulago Hospital in Uganda. A pre-tested self- administered and semi- structured questionnaire was used to collect the data. Data was entered into SPSS and descriptive statistics run on all the variables.

**Results:**

Of the 120 questionnaires distributed, 105 (RR = 87.5 %) were returned and completed. The evidence based comforting strategies used for pain management during IV line insertion by the majority of health care professionals were; skin to skin (51 %) and appropriate upright positioning of the child on mother’s lap (69 %). The least used comforting strategies were; allowing the child to suck his thumb or hand (70 %), use of distraction (69 %) and directing the child to suck one of his fingers into his mouth (90 %). The identified barriers to implementing comforting strategies were; lack of time (42 %), having emergency situations (18 %), and not knowing the right method to use (11 %). Of 105, 100 (95 %) reported that there is need for continuous professional development on comforting strategies.

**Conclusions:**

Findings demonstrated that fewer health care providers used some evidence based comforting strategies of pain relief during pediatric peripheral IV line insertion. Distraction and other evidence based strategies for pain and distress relieve are less often used by the majority of the health care providers. Incorporating pediatric pain management content in all health professionals training curricula could improve the current practices for better health outcomes.

## Background

Venipuncture and intravenous (IV) cannula insertions are the two most common procedures and sources of pain in hospitalized children in health care today [1, 2]. Managing pain proactively during IV cannula insertions or venous access is desirable [2]. Although reports show that elimination or relief of pain is an important responsibility of physicians and nurses caring for children [3], studies show that parent participation through their presence and positioning significantly reduce distress when starting an IV in young children [4]. The WHO asserts that, pain relief is a basic human fundamental right [5, 6] and requires a multidisciplinary approach [1, 6]. Like the adult patients, unmanaged or poorly managed pain in children can result in a variety of negative long-term consequences [5, 7].

Currently, the general scientific community supports the understanding of infant perception of pain and other noxious stimuli [7]. There is considerable evidence that shows a significant proportion of children undergoing venipuncture experience moderate to severe pain as well as elevated levels of pre-procedural and procedural distress [6, 7]. Evidence based non-pharmacological pain management strategies such as distraction strategies are feasible and implemented by all health care providers [3]. Non- pharmacological interventions for pain relief in infants are those that focus on creating a favorable environment, offering pleasant stimuli and centered on maternal care [8].

Health care professionals performing any kind of venipuncture or intravenous cannula insertion procedure should optimize the use available clinical practice guidelines. The used guidelines should advocate for comforting strategies to ameliorate pain and distress related to venous access procedures in children [1, 9–11]. Maintaing a friendly environment when performing painful procedures is imperative [8, 9]. Although there is a growing body of knowledge about nonpharmacological interventions use to increase comfort of children during painful procedures [8, 9, 12], their implementation to date in low resource settings such as Uganda remains very limited [13]. Nonpharmacological strategies are documented as cost effective and easy to administer without formal regulation [8].

Currently, Challenges faced by Uganda include low human resources for health, a growing population, double disease burden of infectious and non-communicable diseases as well as limited access to free published practice guidelines [14]. Nonpharmacological comforting interventions performed by health care providers are the best choice for their additional benefits for better outcomes [8] especially in childhood routine immunizations in primary health care arena.

Numerous reports have documented barriers to implementing pain management strategies for pediatric venous access. These include; attitudes of society as regards to pain perception [15], misconception about pain in children [15], inadequate knowledge [1], underuse of pain assessment tools [16], and lack of recognized standards and guidelines for pain relief [1, 17]. Additionally, lack of family-centered care where family member is not always encouraged as well as not teaching parents what to do to help the child is some of the reported barriers to implementing pain management strategies for pediatric venous access [1, 17].

To our knowledge and based on the needs assessment conducted in Uganda in 2011 [18], none of the current health care providers’ training curricula used, prepares health care professionals to manage pediatric pain. Additionally, there is little no existing guidelines available to direct procedural comfort management by health care providers on the pediatric wards in Mulago National Referral Hospital. Furthermore, there is no documented report available on health care providers’ use of any evidence based interventions for pediatric pain management during peripheral IV line insertion procedures putting children at risk for suboptimal procedural comfort management [17, 19]. This study aimed at establishing the current practices regarding the use of comforting strategies for pediatric pain management by Ugandan health care providers during peripheral IV line insertion procedure. In addition, to determine the barriers impeding the implementation of evidence based comforting strategies to pain management during IV line insertion identified.

## Methods

### Study design and setting

This was a cross sectional and descriptive study involving a convenient sample nurses, doctors and interns (*N* = 105) working in six (6) pediatric wards with the help of a semi- structured self-administered questionnaire designed by the authors. The study was conducted between December 1, 2012 and February 28, 2013 at Mulago Hospital. Mulago hospital is a largest Uganda’s national referral and teaching hospital with a bed capacity of 1500. The hospital is organized into specialization units/departments and among these is pediatric department where the study was conducted. The pediatric and child health care department has more than 6 large units organized into wards: infectious (Jellife), noninfectious (Stanfield), pediatric surgery (2C), pediatric intensive care unit (SCU), acute care unit, nutrition and rehabilitation (Mwanamujimu), Pediatric cancer (LTC), well as the young child clinic (YCC). Each of the pediatric ward has a bed capacity of 50–100 beds depending on the size of the building. The study participants were from 6 pediatric wards.

### Participants and recruitment

In order to obtain the required sample (*N* = 105), the eligibility criteria for inclusion in the study were set; having worked in a pediatric unit for at least no less than 3 months and having performed peripheral IV cannula insertion. The study received approval from the Makerere University Research Ethics Committee (REC: REF 2012–055), as well as obtaining permission from the Department of Pediatrics and Child health, Mulago Hospital. Doctors, nurses and interns are health care providers who participated in the study after consent obtained.

### Data collection

Health care providers who consented and met the eligibility criteria received a self-administered semi-structured questionnaire given by three of the trained research assistants (RAs). The research assistants collected the completed questionnaires from the participants. The developed questionnaire with the use of literature review identified health workers’ comforting strategies used during peripheral intravenous line insertion. The questionnaire consisted of seven (7) demographic characteristics questions: education background (professional cadre), sex, age, tribe, work experience, and employment status. Another set of 18 questions related to their experience using comforting strategies during the IV line insertion procedure were given. This section elicited “yes” and “no” responses where a yes meant that the participant had used that comforting strategy and no, meant having not used that particular strategy or intervention. Three (3) questions asked participants about the need for continuous professional development in IV line insertion and use of comforting strategies as well as a self-rating question on their skills in IV line insertion. The last section on perceived barriers was a multiple responses question that solicited the participants’ opinions on why they did not use any of the comforting strategies during pediatric IV line insertion. Participants were asked to indicate impediment factors using the options provided and if any other to fill in the space provided.

Before actual data collection, two pediatric IV line insertion experts reviewed the questionnaire and their input incorporated in the final version. A pilot study was conducted and consisted of 10 health care providers (5 doctors and 5 nurses) from another nearby hospital pediatric unit and based on their responses and feedback, the questionnaire was finalized for use. Thirty (30) questions were retained on the final version and the average estimated time to complete the questionnaire was 45 min.

### Sample size estimation

Using Kish Leslie (1965) formula: *n* = z^2^pq/e^2^, where *p* = proportion of health care professionals using comforting strategies for pediatric IV line insertion (50 % - estimated as no study available in Uganda on the subject), e = desired level of precision of 5 %, z = 95 % CI (1.96). Sample size required would be 384 participants. Since the number of doctors and nurses working in Mulago pediatric wards is small, all of were individually approached to participate in the study to achieve a total sample of 105 participants.

### Analysis

Data collected was entered and analyzed using Statistical Package for Social Sciences (SPSS version 12). Descriptive statistics was run on all the variables including demographics to identify and exclude missing data. Frequencies and percentages were obtained to guide in the interpretation of the results.

## Results

### Demographic characteristics

Of 120 questionnaires distributed, 105 (RR = 87.5 %) were returned and completed. All the participants (*N* = 105) who participated in the study worked in mainly the 6 (Six) pediatric units or wards, with a mean age of 31 years (ranging from 23 to 60 years) and a mean working experience of 10 years (ranging from 2 to 30 years). Majorities (74 %) of the participants were female health care workers and almost an equal number (50 %) had permanent employment at the hospital (Table 1).

**Table 1:** Socio-demographic characteristics of the study participants

Variable	Frequency (*N* = 105)	Percentage (%)
Sex		
Female	78	74
Male	27	26
Religion		
Catholic	34	32
Moslem	7	7
Protestant	39	37
Others^a^	25	24
Age		
≤30 years	44	42
>30 years	61	58
Tribe		
Ganda	48	46
Nkore	12	11
Others	45	43
Professional Cadre		
Medical Officer	17	16
Senior Housing Officer (SHO)	22	21
BSN	22	21
Diploma nurse	30	29
MSN	1	1
Certificate nurse	3	3
Others^b^	10	10
Years of Working experience		
>3 m < 1 year	24	23
1–3 years	12	11
4–6 years	18	17
7–10 years	17	16
More than 10 years	34	32
Employment status		
Intern (doctors and nurses)	31	30
SHO	21	20
Permanent employee	53	50
Other	0	0

*MSN* Masters of Science in Nursing Degree, *BSN* Bachelors of Science in Nursing Degree, *SHO* Senior House Officer

Others^a^ = Pentecostal, Seventh Day Adventist, Atheist and Jehovah witness

Others^b^ = Nursing Aids, senior student nurses

### Comforting strategies used by health care workers during peripheral IV line insertion

Comforting strategies for pain management mostly used by health workers during peripheral IV line insertion were; greeting the child (72 %) and greeting the parent or care taker (90 %). More than half (51 %) of the Health care providers encouraged skin-to skin contact with the mother, encouraged the mother to breast feed (58 %), positioned the child appropriately on mothers lap in upright position (85 %) but also in addition, ensured that the chosen position was comfortable (69 %) and obtained consent from mother or child (57 %) (Table 2).

**Table 2:** Comforting strategies used by health care providers during IV line insertion

Comforting strategy	Frequency	Percentage (%) *N* = 105
Greeted the child		
Yes	75	72
No	30	29
Greeted the parent/care taker		
Yes	94	90
No	11	10
Allowed child to suckle preferred thumb and or hand		
Yes	31	30
No	74	70
Directed one of the child’s fingers into its mouth for sucking		
Yes	10	10
No	95	90
Avoided the hand the child favored to use		
Yes	47	45
No	58	55
Encouraged skin-to-skin contact with mother		
Yes	54	51
No	51	49
Encouraged the mother to breast feed her child		
Yes	61	58
No	44	42
Explained procedure to the child and gave opportunity to ask questions		
Yes	44	42
No	61	58
Directed the mother to tuck her child		
Yes	40	38
No	65	62
Directed the mother to hand-swaddle her child		
Yes	41	39
No	64	61
Obtained verbal consent from child and mother		
Yes	60	57
No	45	43
Consulted and offered the child the preferred choice of pain relief		
Yes	6	6
No	99	94
Established distraction techniques		
Yes	33	31
No	72	69
Positioned the child appropriately		
Yes	89	85
No	16	15
Allowed the child freedom to select its chosen position		
Yes	29	28
No	76	72
Ensured that the chosen position was comfortable		
Yes	72	69
No	33	31
Provided play preparation like a doll		
Yes	19	18
No	86	82
Consulted the child and mother on previous successes and failures		
Yes	39	37
No	66	63

On the other hand, the least used comforting strategies by the majority of health care providers in pediatric pain management were; allowing the child to suck his thumb or hand (70 %), directing one of the child’s fingers into its mouth for sucking (90 %), allowing the child to have freedom to select the chosen position (72 %) and use of distraction techniques (69 %). A good number (82 %) of health care providers did not provide a play preparation like a doll nor consulted the child or mother on previous successes and failures of IV insertion (63 %) (Table 2).

Furthermore, less than half of health care providers (47 %) responded that there was no preparation in their respective health programs in regard to IV line insertion. Half of the health care providers (50 %) rated themselves “good” at IV line insertion skill. The majority (95 %) reported that continuous professional development on comforting strategies for pediatric pain management was necessary (Table 3).

**Table 3:** Self-rating on use of comforting strategies and IV line insertion skills

Variable	Frequency	Percentage (%) *N* = 105
Health program prepared me in IV line insertion		
Yes	56	53
No	49	47
Self –rating on IV line insertion skills		
1. Excellent	11	10
2. Very good	40	38
3. Good	53	50
4. poor	1	1
Continuous professional develop needs on comforting strategies during IV line insertion		
Yes	100	95
No	5	5

### Perceived barriers to implementing comforting strategies for pain management during IV line insertion procedure

**Fig. 1:**
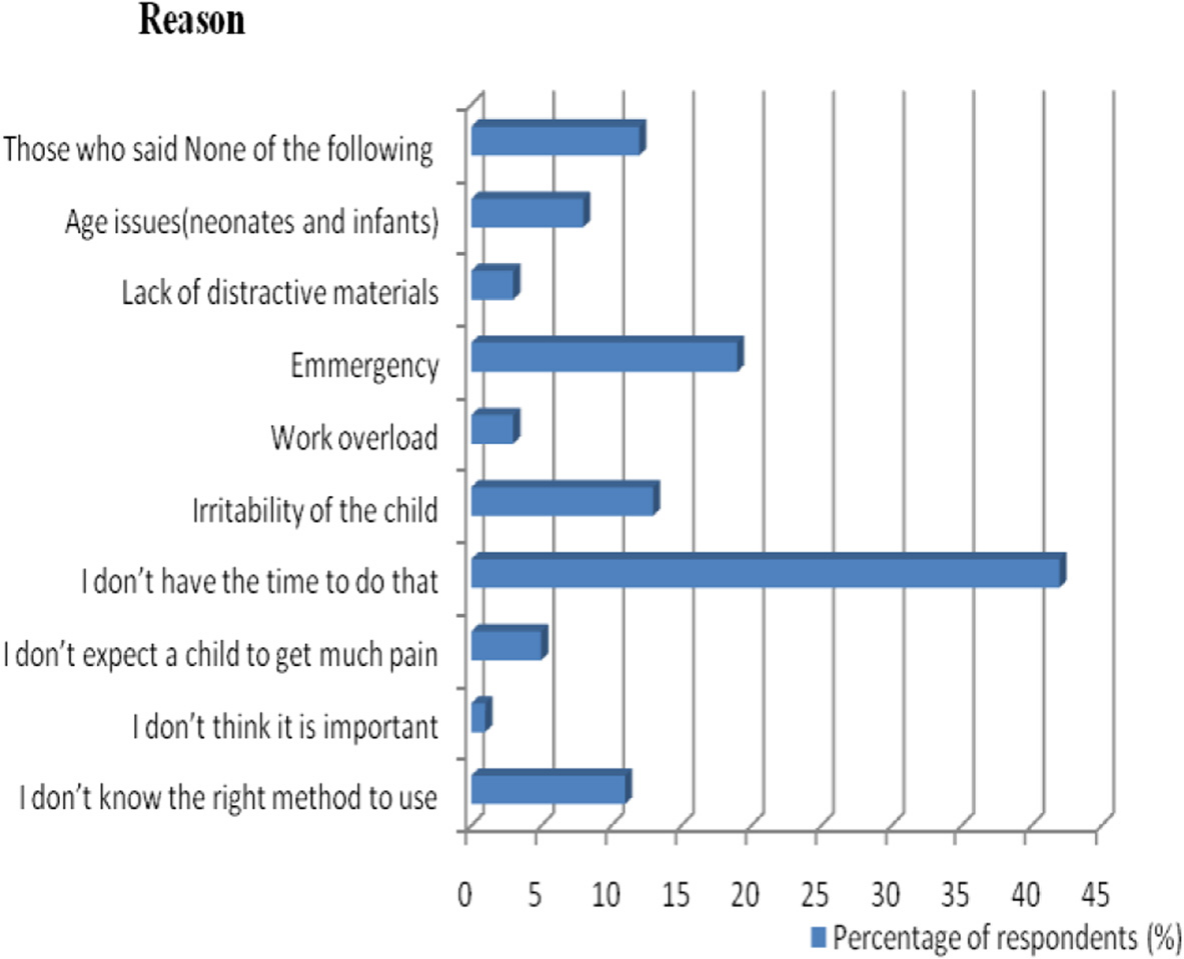
Barriers to implementing comforting strategies for pain management during IV line insertion procedure

A number of barriers that impeded implementation of comforting strategies for pain management during IV line insertion procedures in children were identified. The majority of the participating health care providers responded lack of time for implementation the strategies (42 %), having an emergency situation and encounter (18 %), and irritability of children (13 %). Additionally, health care providers identified not knowing the right method to use (11 %) as an implementation barrier (Fig. 1). Other barriers identified were; age related, work overload, not expecting children to get much pain, lack of distractive materials as well as not thinking that it was important to control pain in this vulnerable group (Fig. 1).

## Discussion

Generally, pediatric pain management remains a huge challenge to all health care providers in primary, acute and other settings in Uganda. It is even more challenging for low resource settings such as Mulago hospital where pain management is not a fundamental right for all patients regardless of the available resources. For this reason, the WHO continues to emphasize pain management as a fundamental right regardless of age, culture, race, ethnicity and socio economic status [5, 6].

Although there is increased awareness of pain management in adult patients [20, 21], pain management in pediatric patients should be given a high priority by all health care professionals through advocating for optimal use of comforting strategies that reduce pain distress during painful procedures [3, 21]. However, our study demonstrated the use of some evidence based comforting strategies for pediatric pain management during IV line insertion by the participating health care professionals at the Mulago national referral and teaching hospital.

Considerably not evidence based comforting strategy for pediatric pain relief; health care professionals reported greeting a child and relative or care taker as a comforting strategy of pain management during venous access procedure. In this study, greeting is an important facet for enhancing rapport and creating a friendly environment that begins with a warm greeting. The finding is supported by findings of two different studies that recommended creating a friendly environment for painful procedures [8, 9]. Conversely, study findings demonstrated that, health care professionals are not aware of the evidence based comforting interventions for pediatric pain management. This could be simply because they have limited access to evidence based guidelines that describe these strategies. Limited access to evidence based guidelines is a major cause of practice variability as found in one study conducted at the same hospital about hypertension management guidelines [14].

Using current evidence practice guidelines has benefits of improving patient care and outcomes [8]. Moreover, making clinical guidelines accessible and available for use enhances current practice for improved patient outcomes but also, plays a vital role in advocating for their use [13, 14]. Complex organizations such as Mulago hospital have a duty to source or develop guidelines in order to regulate or inform practitioners of the current practices. The WHO regularly publishes free online practice guidelines adaptable in low resource settings [14]. Undeniably, procedural pain management guidelines for use in children are necessary for improve procedural outcomes [2]. Notwithstanding, unmanaged pediatric pain is a cause of life threatening mental illnesses such as post-traumatic stress disorders [22]. In addition,,many studies report the potential benefits of managing pain and distress in children; reducing subsequent morbidity and preventing delayed healing [23]. Unfortunately, evaluation of our study against other studies other studies in Africa and Uganda is a challenge due to paucity of studies to compare good practices in pediatric pain management. Studies conducted in Uganda, mostly on the skin-to-skin or kangaroo method provide evidence of the kangaroo method as one that promotes growth and development as well as bonding in preterm babies [24]. Still, in Uganda, little or nothing is documented about kangaroo method as a comforting strategy for pain relief during IV line insertion. But, this study with support from a systematic review, provides evidence for the kangaroo method as efficacious in improving pain reactivity when the caregiver places the infant on the bare chest before, during and after a painful procedure [12]. No wonder, slightly higher than half of the health professionals used skin-to-skin method in the context of kangaroo but underscored the use of tucking and swaddling methods for pain relief. Studies support tucking and swaddling efficacious in reducing pain related distress [12, 25].

Despite the lack of available evidence based guidelines on pediatric pain management during IV line insertion procedure, health care providers’ implemented some comforting strategies available to them. The lack of evidence based guidelines for use by the health care professional partly explains why fewer health care providers used less of the evidence based comforting strategies. In fact, half of the health care providers confessed that health training programs had not prepared them for the skill of pediatric IV line insertion and therefore, majority identified the dare need for continuous professional development about comforting strategies for pediatric pain management.

Other evidence based comforting interventions underutilized by health care providers were; use of destructions techniques and asking a mother or child about previous success or failure of IV line insertion. This finding could be interpreted that health care providers simply do not involve patients in planning their management [26]. It should be noted that parental involvement including; positioning, being present and use of distraction techniques are efficient methods in reducing pain distress in children [12, 26, 27]. Health care professional understanding that different comforting strategies are efficient for different ages with appropriately use when needed is paramount [12, 27].

In our study, the barriers to using evidence based comforting strategies are identified. Specifically and interestingly, participating health care providers reported that they did not have time to implement these strategies. Health care providers reported that having an emergency situations, children being irritable, and not knowing the right methods to use are impeding factors to implement comforting strategies. These are similar to those identified in a study by Zempsky [1]. It is worth noting that the identified barriers significantly affect the provision of quality health care in low resource settings such as Mulago hospital and should be addressed. Additionally, the barriers add to existing challenges of low human resources for health [14].

As a limitation, we acknowledge that our study was limited by the sampling technique and the small number of participating health care professionals. Therefore, results cannot be generalizable. The study also faced the challenge of scarcity of literature from Africa and Uganda and therefore, inference with low-income settings was a challenge. Lastly, the authors depended on self-report information obtained through questionnaire.

### Implications for practice

Evidence based nonpharmacological interventions for pain management during IV line insertion are feasible and cost effective for all health care professions to administer [3, 8]. Nurses are the first persons to have encounters with the patients including children in all health care settings. Again, nurses also perform the majority of the patient procedures including treatment, immunizations and IV line insertions. Being aware of comforting strategies for pain management is an important part in nurses’ job. However, the lack of guidelines may compromise their functionality in the health care team especially when it comes to pain management in children. For Uganda’s case, developing clinical guidelines and disseminating them widely is diserable to address the existing practice gaps among health care providers. Adapting existing guidelines for use by health care professionals is a starting point. Additionally, there is need to enhance collaborative teamwork with a focus on family centered care approach to pain management. Further research to determine the acceptance level of health care professionals in the use of guidelines for pain management that focus on venous access procedures warrants investigation.

### Implication for health professional education

Health professional education that focuses on pain management in children is desirable. Integrating this important subject area in the existing health professions curricula is imperative for practice change. There is need for continuous profession development for all staff rotating in pediatric units about pain management strategies for pediatric patients. Implementing such educational program as part of the orientation program during pediatric rotation for interns, senior house officers, nurses, residents and other health care professionals is essential.

## Conclusions

In conclusion, findings showed that fewer health care providers used some evidence based comforting strategies of pain relief during pediatric peripheral IV line insertion. Distraction and other evidence based comforting strategies for pain and distress relieve are less often used by the majority of health care providers. Seemingly, lack of time, and work overload were major barriers identified to implementing comforting strategies of pain management during pediatric IV line insertion. Incorporating pediatric pain management content in all health professionals training curricula could improve the current practices for better health outcomes.

## Abbreviations

SPSS: Statistical Package for Social Sciences
IV: Intravenous
N: Total number of participants
REC: Research Ethics Committee
REF: Reference Number
